# Molecular Subtyping of Serous Ovarian Tumors Reveals Multiple Connections to Intrinsic Breast Cancer Subtypes

**DOI:** 10.1371/journal.pone.0107643

**Published:** 2014-09-16

**Authors:** Jenny-Maria Jönsson, Ida Johansson, Mev Dominguez-Valentin, Siker Kimbung, Mats Jönsson, Jesper Hansen Bonde, Päivi Kannisto, Anna Måsbäck, Susanne Malander, Mef Nilbert, Ingrid Hedenfalk

**Affiliations:** 1 Division of Oncology-Pathology, Department of Clinical Sciences, Skåne University Hospital, Lund University, Lund, Sweden; 2 CREATE Health Strategic Center for Translational Cancer Research, Lund University, Lund, Sweden; 3 Department of Pathology, Clinical Research Centre, Hvidovre Hospital, Copenhagen University, Hvidovre, Denmark; 4 Division of Obstetrics and Gynecology, Department of Clinical Sciences, Skåne University Hospital, Lund University, Lund, Sweden; 5 Clinical Research Centre, Hvdiovre Hospital, Copenhagen University, Hvidovre, Denmark; Garvan Institute of Medical Research, Australia

## Abstract

**Objective:**

Transcriptional profiling of epithelial ovarian cancer has revealed molecular subtypes correlating to biological and clinical features. We aimed to determine gene expression differences between malignant, benign and borderline serous ovarian tumors, and investigate similarities with the well-established intrinsic molecular subtypes of breast cancer.

**Methods:**

Global gene expression profiling using Illumina's HT12 Bead Arrays was applied to 59 fresh-frozen serous ovarian malignant, benign and borderline tumors. Nearest centroid classification was performed applying previously published gene profiles for the ovarian and breast cancer subtypes. Correlations to gene expression modules representing key biological breast cancer features were also sought. Validation was performed using an independent, publicly available dataset.

**Results:**

5,944 genes were significantly differentially expressed between benign and malignant serous ovarian tumors, with cell cycle processes enriched in the malignant subgroup. Borderline tumors were split between the two clusters. Significant correlations between the malignant serous tumors and the highly aggressive ovarian cancer signatures, and the basal-like breast cancer subtype were found. The benign and borderline serous tumors together were significantly correlated to the normal-like breast cancer subtype and the ovarian cancer signature derived from borderline tumors. The borderline tumors in the study dataset, in addition, also correlated significantly to the luminal A breast cancer subtype. These findings remained when analyzed in an independent dataset, supporting links between the molecular subtypes of ovarian cancer and breast cancer beyond those recently acknowledged.

**Conclusions:**

These data link the transcriptional profiles of serous ovarian cancer to the intrinsic molecular subtypes of breast cancer, in line with the shared clinical and molecular features between high-grade serous ovarian cancer and basal-like breast cancer, and suggest that biomarkers and targeted therapies may overlap between these tumor subsets. The link between benign and borderline ovarian cancer and luminal breast cancer may indicate endocrine responsiveness in a subset of ovarian cancers.

## Introduction

Epithelial ovarian tumors constitute a heterogeneous group of neoplasms that differ in epidemiology, genetic risk factors, precursor lesions and clinical behavior. The different histopathologic subtypes, i.e. serous, mucinous, endometrioid, clear cell and transitional carcinomas and carcinosarcomas, likely have different origins and appear to evolve along distinct pathways [1]–[3]. Alongside with the standard taxane and platinum based agents used for ovarian cancer [4]–[6], multiple targeted agents are being evaluated, with e.g. bevacizumab recently being included in the therapeutic arsenal [7]. Personalized therapy is called for in ovarian cancer particularly since the histopathologic subtypes, as well as tumors with different malignant potential and tumor grade, can be viewed as separate diseases with differences related to both prognosis and treatment response [8]–[12]. Refined molecular subtyping and recognition of key genetic mechanisms constitutes an encouraging basis for further development of subtype-specific targeted therapies.

Previous efforts to characterize ovarian cancers at the molecular level have identified distinct profiles related to the histologic subtypes and have suggested predictive gene signatures [13]–[17]. Tothill *et al.* suggested six different subtypes, referred to as C1–C6, based on serous and endometrioid ovarian, primary peritoneal and fallopian tube tumors. The C1–C2 and C4–C5 subtypes, in general, are thought to characterize high-grade serous tumors. The C1 signature is characterized by a high degree of desmoplasia, C5 by mesenchymal genes and overexpression of proliferation genes and the C2 and C4 signatures by high numbers of intra-tumoral and stroma associated CD3+ cells. The signatures are outcome predictive, with the C1 signature corresponding to a considerably worse outcome than the other signatures. The C3 signature represents low-grade serous and borderline tumors and the C6 signature low-grade, early-stage endometrioid tumors; in general they show good response to treatment and long-time survival [18]. Likewise, molecular subtyping in breast cancer is well established and recent reports have recognized similarities between high-grade serous ovarian cancer and basal-like breast cancer [19].

We performed global gene expression profiling of serous ovarian tumors, including serous cystadenomas, serous borderline tumors and serous adenocarcinomas, and applied previously described gene signatures including the well-known intrinsic breast cancer subtypes [18], [20]–[22] to outline further possible similarities between these tumor types. Since mutations in the MAPK/ERK pathways are common in both borderline and low-grade ovarian cancer and luminal breast cancers the presence of *KRAS* and *BRAF* mutations was investigated among the ovarian tumors [23], [24]. Shared common features between ovarian and breast cancer may be useful for future development of predictive biomarkers and tailored treatments in both tumor types, and in this study we present interesting connections between the molecular subtypes of ovarian and breast cancer.

## Materials and Methods

### Tumor samples

In total, 37 serous ovarian adenocarcinomas, 17 serous cystadenomas/adenofibromas and 5 serous borderline tumors were obtained from the Skåne University Hospital ovarian tumor biobank (table 1). A total of 13 biological replicates (6 omental metastases, 1 pelvic metastasis and 2 metastases to the contralateral ovary as well as 3 benign and 1 borderline ovarian tumors) were included to account for intra-tumor heterogeneity. All tumor samples were collected at primary surgery (2003–2011) and the patients had not received chemotherapy prior to surgery. Histologic subtype and grade were determined according to Silverberg and WHO [25], [26] and all tumors were staged according to the International Federation of Gynecology and Obstetrics (FIGO) criteria. Hematoxylin & Eosin stained slides were used to assess tumor grade. This was performed by a senior pathologist (AM). Ethical approval for the study was granted from the Lund University ethics committee, Sweden, waiving the requirement for informed consent for the study.

**Table 1:** Clinicopathologic features of malignant and borderline ovarian tumors in the study cohort.

Id	Feature	Stage	Grade	Age at diagnosis (years)	Tissue type	Survival (years)	C- signature	BC subtype
38	M	IIIC	3	69	Ovary	3	C1	Normal
70	M	IIIC	3	82	Omentum	1	C1	Normal
84	M	IIC	2	69	Ovary	6	C5	HER2
90	M	IIIC	2	60	Ovary	2	C3	Normal
106	M	IIIC	3	71	N/A	4	C2	Basal
118	M	IIIC	3	50	N/A	2	C5	Basal
125	M	IIIA	3	71	N/A	5	C4	Basal
137	M	IIA	3	70	FT	Alive	C4	Basal
153	M	IIIC	3	61	Ovary	1,5	C5	Basal
159	M	IIIC	3	91	N/A	<1	C1	Basal
186	M	IIIC	3	82	Cystic fluid	2	C4	Basal
190	M	IIIC	2	79	Omentum	1,5	C5	Luminal A
192	M	IV1	2	55	Omentum	1	C1	Basal
207	M	IIIC	1	80	N/A	2	C1	Luminal A
219	M	IIIC	3	67	FT	1	C1	Basal
225	M	IV1	2	53	N/A	2	C2	HER2
226	M	IV3	3	61	Omentum	Alive	C4	Basal
232	M	IV3	1	87	Ovary	<1	C3	Normal
251	M	IV3	3	59	Omentum	1	C1	Basal
273	M	IIIC	3	62	N/A	4	C2	HER2
275	M	IIIC	2	65	Ovary	2	C1	Basal
279	M	IIIB	2	69	Ovary	1	C1	Basal
284	M	IIIB	3	70	Omentum	Alive	C1	Normal
293	M	IIIC	3	72	N/A	Alive	C1	Basal
297	M	IIIC	3	65	Ovary	2	C1	Basal
305	M	IIIB	2	63	Ovary	2	C2	Basal
306	M	IIIC	1	40	N/A	Alive	C4	HER2
307	M	IIC1	1	42	Ovary	Alive	C2	Basal
311	M	IIB	3	67	N/A	Alive	C2	Basal
314	M	IIIC	2	69	N/A	Alive	C4	HER2
330	M	IC1	3	64	Ovary	Alive	C5	Luminal B
344	M	IIIC	3	68	Ovary	Alive	C4	Basal
393	M	IIIC	3	81	Ovary	3	C2	Basal
397	M	IIIC	2	79	Omentum	Alive	C3	Normal
402	M	IIIC	3	70	Ovary	Alive	C4	Luminal B
420	M	IC1	1	67	Ovary	Alive	C4	Luminal B
438	M	IIIC	3	53	Ovary	Alive	C2	Basal
16	Bo	IA	N/A	40	Ovary	Alive	C3	Luminal A
48	Bo	IA	N/A	51	Ovary	Alive	C3	Luminal A
86	Bo	IA	N/A	45	N/A	Alive	C3	Luminal A
377	Bo	IC1	N/A	67	Ovary	Alive	C3	Normal
385	Bo	IC1	N/A	60	Ovary	Alive	C3	Luminal A

*Feature*: M = Malignant, Bo = Borderline; *Tissue type*: tissue used for RNA extraction, FT = Fallopian Tube, N/A = Unknown; *Survival*: Disease specific survival, Alive = alive at start of study; *C-signature*: corresponding ovarian molecular subtype [18]; *BC subtype*: corresponding intrinsic breast cancer subtype [22].

### RNA extraction and gene expression analysis

Total RNA was extracted using the Allprep kit (Qiagen, Heidelberg, Germany) according to the manufacturer's instructions. RNA concentration was determined using a NanoDrop Spectrophotometer (NanoDrop Technologies, Wilmington, DE) and samples with ≥200 ng RNA with 260/280 ratios ≥1.8 were used for further analysis. RNA quality was assessed using a Bioanalyzer (Agilent technologies, Santa Clara, CA), and RNA integrity numbers (RIN) >6 were regarded as sufficient.

Gene expression profiling analyses were performed at the SCIBLU Genomics Centre, Lund University, Sweden. The cDNA synthesis, labeling, and subsequent hybridization to the HumanHT-12 v4 Expression BeadChips (Illumina Inc., San Diego, CA) was performed according to the manufacturer's instructions. The Illumina HumanHT-12 v4 Expression BeadChips allow genome-wide expression profiling of more than 47,000 gene transcripts and splice variants. The 59 samples and 13 biological replicates were randomized on the chips. The BeadChips were then scanned on an i-Scan (Illumina Inc.), during which fluorescence intensities were read and images extracted. The gene expression data are available in the National Center for Biotechnology Information Gene Expression Omnibus [GEO accession number: GSE57477] [27].

### Data analysis

Gene expression data were uploaded to the GenomeStudio software (Illumina Inc.), quantile normalized, background corrected and log2 transformed. Probes with a mean intensity <2.5 and variance <0.1 were excluded, leaving a total of 16,024 probes corresponding to 12,313 unique genes. Thereafter the data were uploaded to the MeV v4 software, an application used for identification of genes and expression patterns in microarray data [28], mean centered and a variance filter was applied to select the 20% of the probes with the greatest variation of expression across the dataset. Unsupervised hierarchical clustering was performed using complete linkage and Pearson distant metric. Two-class unpaired significance analysis of microarrays (SAM) was performed based on all 16,024 probes to identify differentially expressed probes between the different tumor subgroups (benign, borderline, malignant) at a false discovery rate (FDR) <0.01 [29]. Hierarchical clustering, supervised by the SAM analysis results and thereby identifying significantly differentially expressed genes between the tumor subgroups, was performed using the same methods as for unsupervised clustering. Gene ontology analyses based on the significant genes were performed in the gene ontology enrichment analysis and visualization tools GOrilla and ToppGene for identification of possible gene enrichment with biological or functional differences separating the subgroups [30], [31].

### Molecular subtyping and external data sets

Gene signatures outlining six molecular subtypes of high-grade and advanced stage serous ovarian tumors as well as endometrioid, low-grade serous and borderline ovarian tumors (referred to as the “Tothill dataset”) [18] were applied to the serous ovarian tumors in our cohort. Data were normalized and log2 transformed using the Gene Chip Operating Software Version 1.4 with Affymetrix default analysis settings. Probes with intensity values <4 and variance <0.15 were excluded. The six ovarian cancer signatures contained in total 4,732 probes of which 4,099 probes, corresponding to 2,725 unique genes, with good quality were left after filtering away probes with bad quality. 1,295/2,725 (47.5%) of these genes were identified in our dataset and used for further analyses. To validate the classifier, the 1,295 genes present in our dataset were re-applied to the 285 ovarian tumors in the original cohort, thereby re-assigning subtypes to each tumor. A gene signature for intrinsic subtyping of breast cancer was also applied to our serous ovarian tumors [22]. Each tumor in our cohort was classified into the molecular subtypes of ovarian cancer (C1–C6) as well as the intrinsic subtypes of breast cancer (luminal A, luminal B, basal-like, normal-like, and HER2 enriched) using nearest centroid classification. The methodology for nearest centroid classification is outlined by Johansson *et al*.[32]. Validation was performed by classifying the 285 ovarian tumors in the Tothill dataset into the intrinsic breast cancer subtypes. Furthermore, seven gene expression modules representing key biological processes in breast cancer (AURKA/proliferation, CASP3/apoptosis, ERBb2/HER2 signaling, ESR1/ER signaling, STAT1/immune response, PLAU/tumor invasion and metastasis, VEGF/angiogenesis; referred to as the “Desmedt modules”) were applied to the serous ovarian tumors in our dataset as well as the Tothill dataset [33], and their relationship to the previously described intrinsic breast cancer subtypes was investigated. These modules, derived from 917 breast cancers in publicly available datasets and characterized by computed module scores, comprise in total 889 genes.

### Mutation analysis

KRAS mutation analysis was performed using the Roche cobas K-RAS Mutation Kit (product number 05852170190) (Roche, Pleasanton, CA), a CE-IVD real-time melting curve KRAS mutations assay, detecting mutations in codons 12, 13 and 61 of the KRAS oncogene. BRAF mutation analysis was performed using the Roche cobas BRAF V600 mutational analysis (product number 05985595190), which evaluates the BRAF V600 site in exon 15 and detects wildtype or mutated V600. The analyses were performed according to the manufacturer's instructions, and the assays were run on the z480 Lightcycler (Roche). The mutation analyses were performed at the Department of Pathology, Clinical Research Centre, Hvidovre Hospital, Denmark.

### Statistical methods

Mann-Whitney U-test and Pearson correlation were used for comparison between expression profiles of the different tumor subsets using the MeV 4.6.02 software. Correlations between different subtype classifications were assessed using Fisher's exact test and between module scores using Mann-Whitney U-test and Kruskal Wallis test in SPSS (IBM SPSS Statistics 19). P-values <0.05 were considered statistically significant.

## Results

### Comparison of malignant, benign and borderline serous ovarian cancers

Unsupervised hierarchical clustering of benign and malignant tumors based on the 20% of the probes that showed the most variability revealed two distinct clusters, one containing only malignant tumors and one containing all benign and four malignant tumors (Figure S1). All but one of the biological replicates clustered together pair-wise. The clusters remained stable after removal of the biological replicates, suggesting stable transcriptional differences between the clusters (Figure S2). Of note, of the four malignant tumors in the benign cluster one was grade 1, two were grade 2 and one was grade 3, but no significant differences regarding stage or mean age at diagnosis were seen between the malignant tumors in the two clusters.

**Figure 1:**
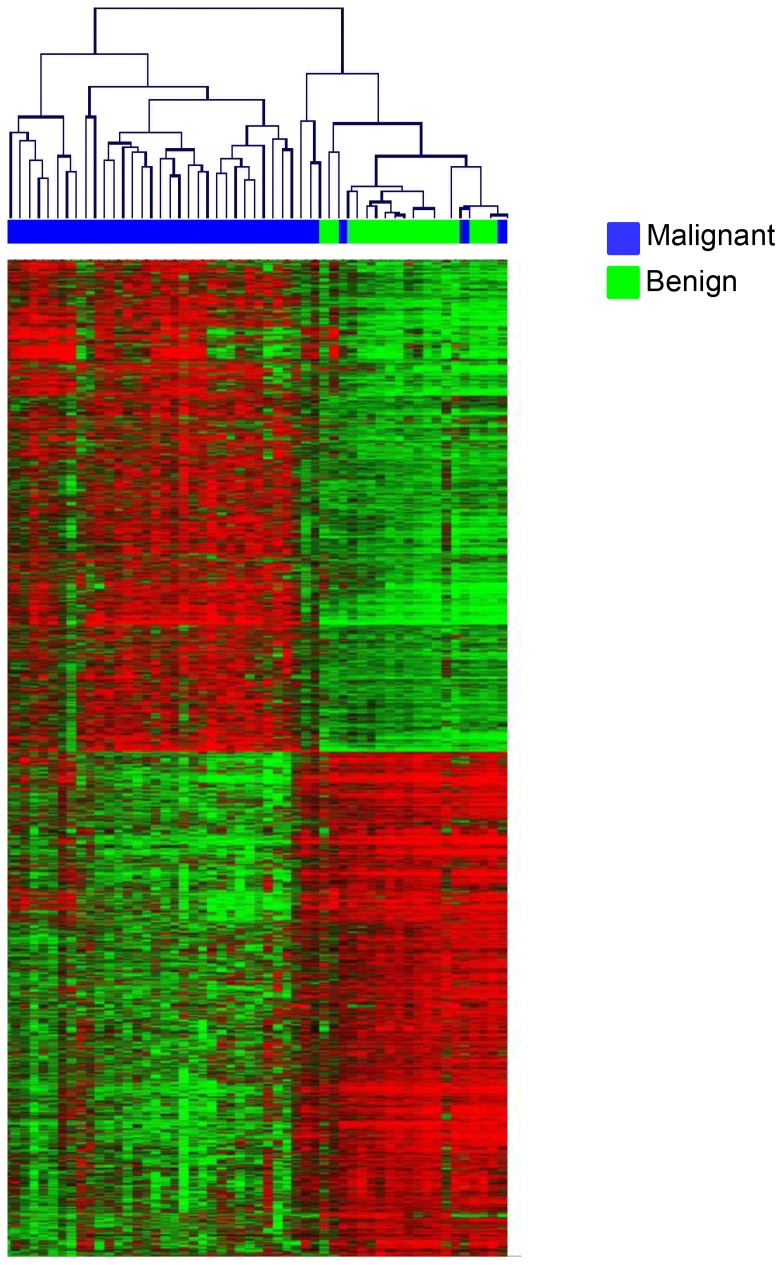
SAM analysis. Supervised hierarchical clustering of malignant (n = 37) and benign (n = 17) serous ovarian tumors (FDR <1%). Red represents relative upregulation and green represents relative downregulation.

Next, a SAM analysis was performed to explore transcriptional differences between benign and malignant tumors, revealing 5,944 significantly differentially expressed genes (FDR <0.01), of which 2,984 were upregulated and 2,960 were downregulated among the malignant tumors (figure 1, Table S1).

Cell cycle kinases (e.g. *CDC2, CDC5, CDC7* and *CDC20*) as well as *AURKA* and *S100A9*, which can all broadly be linked to cell cycle regulation and mitosis, were upregulated in the malignant tumors. Consistent with this, gene ontology analyses revealed a significant upregulation of cell cycle associated biological processes (ToppGene, FDR<0.05; table 2).

**Table 2:** The 10 most significantly enriched biological processes in the malignant ovarian tumors in the study cohort [31].

	Biological process*	p-value	Genes from input^a^	Genes in annotation^b^
1.	mitotic cell cycle	3.823E^−24^	213	874
2.	cell cycle process	1.544E^−23^	265	1192
3.	antigen processing and presentation ofexogenous peptide antigen	2.768E^−19^	67	171
4.	antigen processing and presentation ofexogenous peptide antigen viaMHC class I	3.943E^−19^	43	80
5.	cellular response to stress	6.028E^−19^	279	1370
6.	symbiosis, encompassing mutualismthrough parasitism	1.886E^−18^	175	741
7.	interspecies interaction betweenorganisms	1.886E^−18^	175	741
8.	antigen processing and presentation ofexogenous antigen	3.374E^−18^	67	178
9.	mitotic cell cycel phase transition	5.793E^−18^	121	445
10.	cell cycle phase transition	6.556E^−18^	122	451

*FDR<0.05 and ≥3 recognized genes/biological function were required to consider a gene ontology (GO) process significant. 731 significant.

GO processes were identified.

a Number of genes in the study cohort correlating to the GO process.

b Number of genes in the GO process.

To investigate whether borderline tumors are more closely related to benign or malignant tumors, an unsupervised clustering based on the 20% most varying probes across the dataset, and a hierarchical clustering supervised by the 5,944 significantly differentially expressed genes between benign and malignant tumors, were performed on the whole dataset. The analyses resulted in two distinct clusters, one malignant and one benign. The borderline tumors were split between the two main clusters, implying heterogeneity within this group (Figure S3).

We next applied the gene signatures described by Tothill *et al.* [18] to study the representation of molecular ovarian subtypes (“C-signatures”) in our dataset. Using nearest centroid classification, a specific ovarian cancer C-signature was assigned to each tumor in our cohort. 52/59 (88%) of the tumors had a correlation coefficient ≥0.2. The centroid classifications revealed considerable heterogeneity across the tumors (p<0.001; table 3). These differences prompted us to investigate each signature individually, and significant correlations between the malignant tumors in our cohort and the C1, C2 and C4 signatures (p = 0.020), and between the benign and borderline tumors in our cohort and the C3 signature (p<0.001) were revealed (Table S2). 251/285 tumors in the original Tothill cohort had an assigned C-signature, and 239/251 (95.2%) were correctly re-assigned to their respective C-signatures, thereby validating the classification method (Table S7).

**Table 3:** Ovarian cancer subtypes.

	C-signature					
	C1	C2	C3	C4	C5	Total
**Malignant**	12	8	3	9	5	37
(% within group)	(32.4)	(21.6)	(8.1)	(24.3)	(13.5)	(100.0)
**Borderline**	0	0	5	0	0	5
(% within group)	(0.0)	(0.0)	(100.0)	(0.0)	(0.0)	(100.0)
**Benign**	1	0	15	0	1	17
(% within group)	(5.9)	(0.0)	(88.2)	(0.0)	(5.9)	(100.0)
p<0.001						

Serous ovarian tumors in the study cohort with corresponding ovarian cancer subtypes (“C-signatures”) [18]. The rows outline the tumor types with the representation in each subtype in percent within parentheses. The p-value is calculated using Fisher's exact test.

### Exploring similarities between ovarian and breast cancer

To investigate potential similarities between ovarian cancer and the widely acknowledged intrinsic subtypes of breast cancer, beyond the similarities between high-grade serous ovarian cancer and basal-like breast cancer that have been reported, we applied the signatures representing the intrinsic breast cancer subtypes to our cohort [19], [22]. 40/59 (68%) of the tumors had a correlation coefficient ≥0.2. Classification of the intrinsic breast cancer subtypes was applied to the ovarian tumors in our cohort, revealing considerable heterogeneity (p<0.001; table 4). Significant correlations between the malignant ovarian tumors and the basal-like breast cancer subtype (p<0.001), and between the non-malignant (benign and borderline) ovarian tumors and the normal-like breast cancer subtype were found (p<0.001) (Table S3). The borderline tumors in our cohort, all of which were most highly correlated to the ovarian cancer C3 signature, also had highest correlation to the luminal A breast cancer subtype (p<0.001) (Table S4), thus extending the links between the two tumor types.

**Table 4:** Intrinsic breast cancer subtypes.

	Intrinsic breast cancer subtype					
	Luminal A	Luminal B	Basal-like	Normal-like	Her2	Total
**Malignant**	2	3	21	6	5	37
(% within group)	(5.4)	(8.1)	(56.8)	(16.2)	(13.5)	(100.0)
**Borderline**	4	0	0	1	0	5
(% within group)	(80.0)	(0.0)	(0.09	(20.0)	(0.0)	(100.0)
**Benign**	1	0	0	16	0	17
(% within group)	(5.9)	(0.0)	(0.0)	(94.1)	(0.0)	(100.0)
p<0.001						

Serous ovarian tumors in the study cohort with corresponding intrinsic breast cancer subtypes [22]. The rows outline the tumor types with the representation in each subtype in percent within parentheses. The p-value is calculated using Fisher's exact test.

### Validation of the intrinsic breast cancer subtypes in ovarian cancer

**Figure 2:**
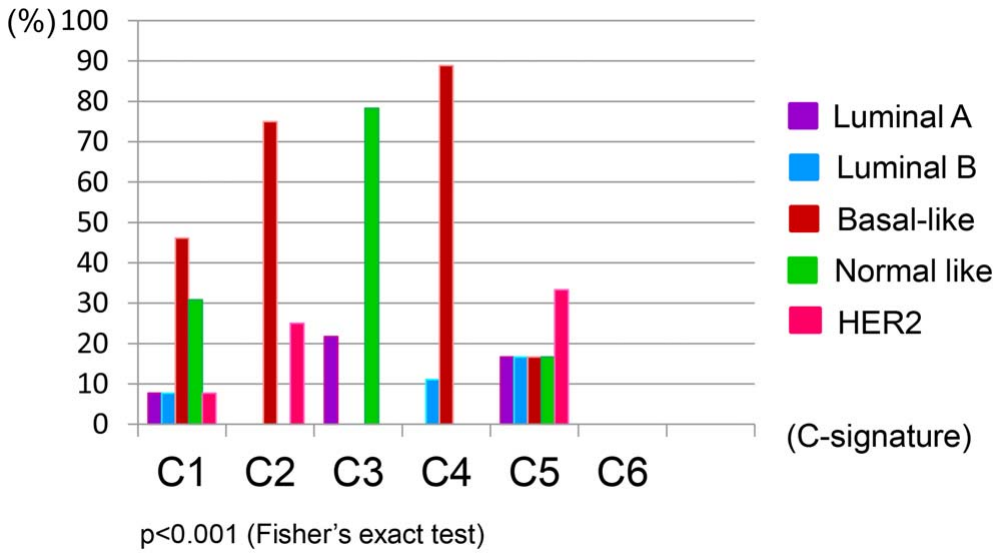
Correlations between ovarian and breast cancer molecular subtypes. Correlations between specific ovarian cancer C-signatures [18] and the intrinsic breast cancer subtypes [22] in the serous ovarian tumors in the study cohort. Tumors within each ovarian cancer C-signature are shown along the X axis, and the colored bars represent the percentage (on the Y axis) of each intrinsic breast cancer subtype within the respective C-signatures.

Next, potential correlations between ovarian cancer C-signatures and the intrinsic breast cancer subtypes were explored. When the C-signatures for the tumors in our cohort were correlated to the intrinsic breast cancer subtypes for the same tumors, a significant heterogeneity within the tumor cohort was observed (p<0.001; figure 2). The ovarian cancer C2 and C4 signatures correlated significantly with the basal-like breast cancer subtype (p = 0.019 and p = 0.001, respectively) (Table S5), while the C3 signature correlated to the normal-like breast cancer subtype (p<0.001) (Table S6). These observations suggest commonalities between the transcriptionally based molecular classifiers of ovarian and breast cancer (Figure S4). The results were verified by classifying the ovarian tumors in the Tothill dataset [18] into the intrinsic breast cancer subtypes; similar links between the C2 signature and the basal-like subtype, and the C3 signature and normal-like subtype were observed (p<0.001; Table S8). Notably, 16/26 patients (61.5%) whose tumors resembled the basal-like and HER2 enriched intrinsic breast cancer subtypes died from their disease within four years from diagnosis, compared to only 3/9 patients (33.3%) whose tumors displayed gene expression profiles corresponding to the luminal A and luminal B intrinsic breast cancer subtypes.

**Figure 3:**
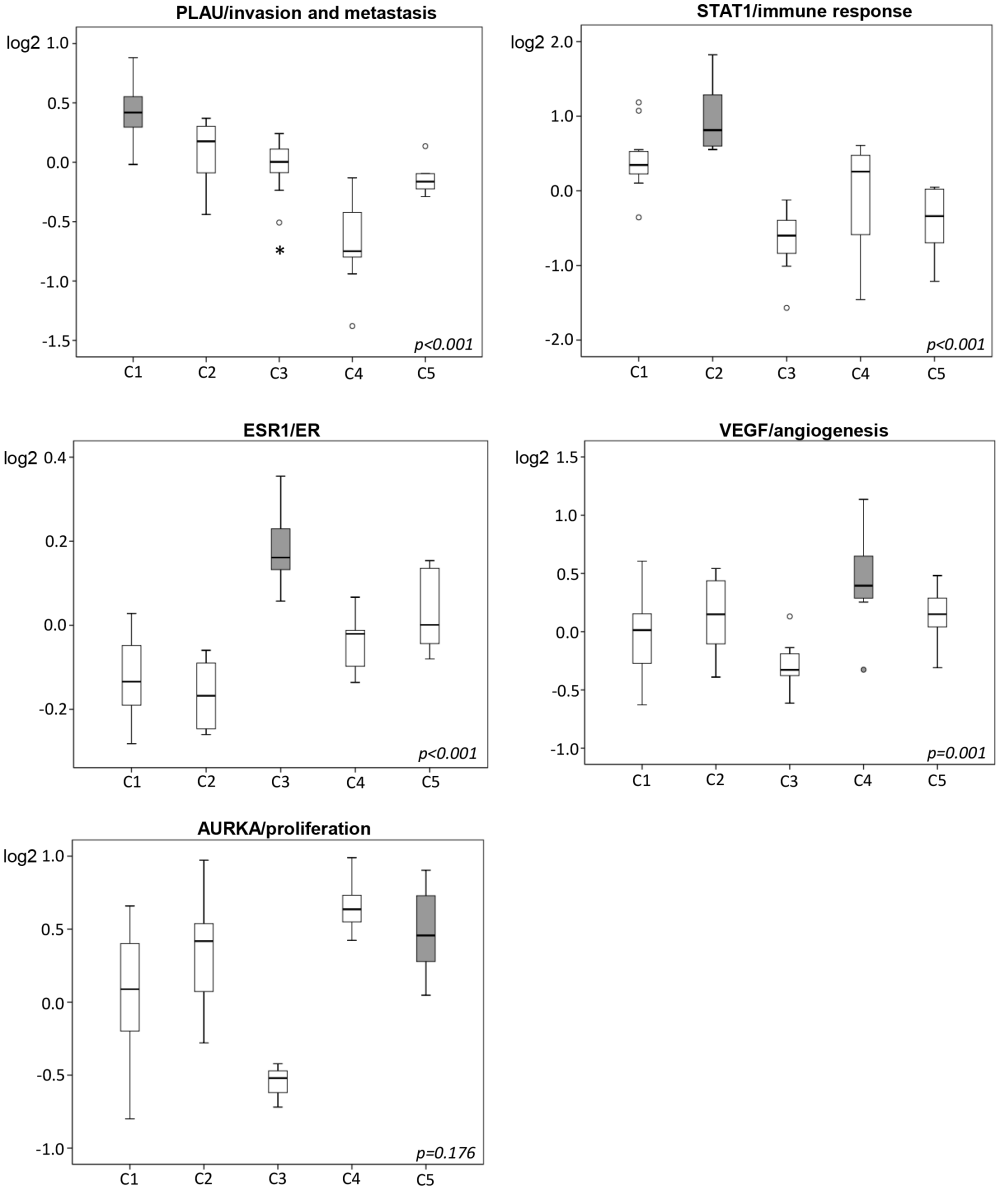
Functional gene expression modules. Correlations between the ovarian cancer C1–C5 signatures and the functional breast cancer modules by Desmedt *et al* [33]. Log2 mRNA values are presented on the Y axis. p-values for the highlighted boxes vs. the rest in each plot are calculated using the Mann-Whitney U Test.

To further explore potential connections between the molecular subtypes of ovarian and breast cancer, we applied the gene expression modules representing key biological features of breast cancer described by Desmedt *et al.* to our tumors [33]. As for the ovarian cancer C-signatures and the intrinsic breast cancer subtypes, considerable heterogeneity within the tumor cohort was found. The malignant tumors displayed a significantly higher module score than the benign and borderline tumors for the AURKA/proliferation, STAT1/immune response, CASP3/apoptosis, VEGF/angiogenesis and ERBb2/HER2 signaling modules. The borderline and benign tumors on the other hand correlated to the ESR1/ER signaling module (Figure S5). The somewhat surprising absence of a correlation between the malignant tumors and the PLAU/tumor invasion and metastasis module led us to investigate the correlation between the Desmedt modules and the ovarian C-signatures. Again, significant differences within the cohort were observed, with highly significant correlations between the C1 signature and PLAU/invasion module (p<0.001), the C2 signature and STAT1/immune response module (p<0.001), and the C4 signature and VEGF/angiogenesis module (p = 0.001). The C5 signature showed a trend towards correlation to the AURKA/proliferation module compared to non-C5 tumors, but did not reach statistical significance (p = 0.176). The C3 signature correlated significantly to the ESR1/ER module (p<0.001, figure 3). Finally, we also applied the Desmedt modules to the tumors in the Tothill dataset, and could verify the correlations described between the ovarian cancer C-signatures and the functional breast cancer derived gene expression modules (Figure S6). In addition, in this larger cohort, the C5/AURKA correlation was also found to be significant (p<0.001).

### Mutation analysis

Mutation analyses revealed four tumors with *KRAS* mutations and two tumors with *BRAF* mutations in our cohort. Four of these mutations were present among the borderline tumors, all of which corresponded to the C3 ovarian cancer signature and the luminal A breast cancer subtype, respectively. Two malignant tumors, both grade 1, harbored mutations in *KRAS* and corresponded to the C3/normal-like subtypes and C4/luminal B subtypes, respectively (table 5).

**Table 5:** *KRAS* and *BRAF* mutations.

Id	Feature	C-signature	BC subtype	Mutation
420	M	C4	Luminal B	KRAS
232	M	C3	Normal	KRAS
86	Bo	C3	Luminal A	KRAS
385	Bo	C3	Luminal A	KRAS
16	Bo	C3	Luminal A	BRAF
48	Bo	C3	Luminal A	BRAF

Distribution of KRAS and BRAF mutations and their correlations to ovarian cancer C-signatures and intrinsic breast cancer subtypes.

*Feature*: M = Malignant, Bo = Borderline; *C-signature*: corresponding molecular ovarian cancer subtype [18]; *BC-subtype*: corresponding intrinsic breast cancer subtype [22].

## Discussion

Serous carcinomas account for about 50% of the malignant epithelial ovarian tumors and thereby constitute the predominant histologic subtype. Type 1 tumors, i.e. low-grade serous carcinomas, along with low-grade endometrioid, mucinous and clear cell tumors, are thought to develop step-wise from benign cystadenomas/adenofibromas via borderline tumors and are typically slowly proliferating and frequently harbor mutations in *KRAS, BRAF* and *PTEN*. In contrast, type 2 tumors (high-grade serous and high-grade endometrioid ovarian tumors, carcinosarcomas and undifferentiated carcinomas) are suggested to develop from precursor lesions in the fallopian tube [24] and are characterized by rapid progression and frequent *TP53* mutations [10], [34]–[36]. Molecular subtyping of ovarian cancer is being increasingly recognized, with e.g. the six transcriptionally based ovarian C-signatures proposed by Tothill *et al.* being both descriptive (with good correlations to clinical factors) and predictive of outcome [18].

Molecular subtypes of breast cancer have been established and linked to clinical behavior and treatment response [20], [21]. Although about 60% of all ovarian tumors display high expression of estrogen receptors (ER) [37], features shared between high-grade serous ovarian cancer and basal-like breast cancer (the majority of which are “triple negative”, i.e. lack expression of estrogen and progesterone receptors and HER2 amplification) have recently been reported. Both tumor groups display frequent *TP53* mutations and genomic instability and are clinically aggressive. Also, *BRCA* mutations are more frequent in high-grade serous ovarian cancers and in basal-like breast cancers in the case of *BRCA1* [19]. The diagnostic and therapeutic potential in clarifying ovarian cancer heterogeneity and identifying mechanisms shared between ovarian cancer and breast cancer constituted the basis of our study.

SAM analysis between the malignant and benign ovarian tumors in our cohort revealed enrichment of cell cycle associated processes among the malignant tumors, in line with malignant tumors being highly proliferative *per se*. Although very few borderline tumors were included in our cohort, the fact that they were divided between the benign and the malignant tumor clusters, regardless of whether the clustering was supervised by significantly differentially expressed genes between benign and malignant tumors, or unsupervised, is in line with other studies indicating that borderline tumors constitute a very heterogeneous group with both benign and malignant features [38].

Classification of the ovarian tumors in our cohort using the C-signatures demonstrated the presence of all but the C6 signature. Since the C6 signature is characterized mainly by low-grade endometrioid tumors, which were not present in our cohort, this finding supports the ability of gene signatures to capture histological differences, and indicates that the C-signatures are stable and widely applicable across datasets and microarray platforms. The malignant tumors correlated significantly to the C1, C2 and C4 signatures, and as anticipated from the recent data reported from the TCGA, classification into the intrinsic breast cancer subtypes also revealed a significant correlation between malignant ovarian tumors and the basal-like breast cancer subtype [19]. This link was further supported by the finding that the ovarian tumors classified as basal-like in our cohort in turn correlated to the C2 and C4 signatures. A majority of both high-grade serous ovarian and basal-like breast cancers express high levels of proliferation genes [16], [19]. Proliferation, among other biological processes, is captured by the Desmedt modules, and we could correlate the C-signatures to these modules and verify the finding in an independent dataset. The C2 “high immune signature” correlated significantly to the STAT1/immune response module, for example, and the C1 “high stromal response signature” to the PLAU/invasion and metastasis module. Taken together, the breast cancer derived Desmedt modules capture the nature of the C-signatures as outlined by both Tothill *et al.* and the TCGA well [16], [18] and provide further biological information regarding the differences in phenotype between the subgroups. Moreover, the statistical correlations shown here further support the link between the subtypes of serous ovarian and breast cancer.

The C3 signature in the original study encompassed borderline (low malignant potential, LMP) tumors and, as expected, the benign and borderline tumors in our cohort correlated significantly to the C3 signature. This signature is characterized by a relative overexpression of genes in the MAPK/ERK pathway, in line with the fact that type 1 ovarian tumors often harbor mutations in *KRAS* and *BRAF*. Four of five borderline tumors in our cohort displayed *KRAS* or *BRAF* mutations, and the two malignant tumors harboring mutations in *KRAS* in turn correlated to the C3 and C4 ovarian signatures. Interestingly, a significant correlation between the few borderline tumors and the luminal A breast cancer subtype was found. Luminal breast tumors frequently display mutations in the MAPK/ERK pathway, thereby resembling the ovarian type 1 tumors (and the ovarian C3 signature). This is further supported by the independent finding of a significant correlation between the ovarian C3 signature and the ESR1/ER signaling breast cancer module by Desmedt *et al.* [33]. Hence, although the borderline tumors in our cohort were interspersed between the malignant and benign neighbors in the clustering analyses, upon comparison with the ovarian cancer C-signatures and the intrinsic breast cancer subtypes, they showed obvious similarities with benign and low-grade malignant tumors – as anticipated based on the prototypic type 1 tumors they are described as. Furthermore, the luminal A and B (estrogen receptor positive) breast cancer subtypes differ in transcriptional profiles, mutation spectra and overall survival [19]; this is in line with the clinical spectrum observed in low-grade serous ovarian cancer, with low-grade, early-stage tumors showing a favorable prognosis, while low-grade, advanced stage tumors tend to respond poorly to chemotherapy. Despite the generally high expression of ER in ovarian cancer, the response to both tamoxifen and letrozole has been limited [39], [40]. The different isoforms of ER seem to vary with the malignant potential, with the beta isoform (ERβ) reported to be less expressed in malignant ovarian tumors compared to borderline tumors and benign ovaries, but whether ERβ or ERα influence outcome is not clear [41], [42]. In contrast, ERα is a favorable prognostic factor in breast cancer [43]. A recent study by the Ovarian Tumor Tissue Analysis consortium (OTTA) focused on expression of ERα and the progesterone receptor (PR), and in a large series only strong expression of PR, but not ERα, was correlated to increased survival in high-grade serous ovarian cancer. No significant correlations between ERα or PR expression and survival were found in multivariate analyses of low-grade serous tumors [44]. The vast majority of the low-grade tumors were however ER and/or PR positive, but the few that were negative did not have a significantly different outcome despite the fact that the majority of the low-grade tumors were stage III–IV. Likewise, ERα is reported to be overexpressed in serous borderline tumors [41]. Taking these results into consideration in light of our findings of a correlation between the ovarian C3 signature and the luminal A breast cancer subtype, it would be interesting to study the response to and the potential effect of endocrine treatment specifically in advanced type I ovarian tumors.

## Conclusions

The findings in this study support that transcriptional signatures indeed capture the biology of transforming events and oncogenic mutations and also support similarities between molecular subtypes of ovarian and breast cancer beyond high-grade serous ovarian cancer and basal-like breast cancer. Though limited series are sensitive to overfitting, importantly, our findings were stable and reproducible in a large independent cohort. The similarities between molecular subtypes of ovarian and breast cancer may be of potential interest for further studies regarding targeted therapies and the use of chemotherapeutic agents in ovarian cancer, as well as biomarker studies. While the proposed similarities between low-grade serous and borderline ovarian (type 1) tumors and luminal breast cancers may in part be attributable to similarities in proliferation rates compared to high-grade ovarian (type 2) and basal-like breast cancers, other biological similarities, such as potential endocrine responsiveness, are thought-provoking and merit further investigation.

## Supporting Information

Figure S1 **Hierarchical clustering.** Unsupervised clustering of malignant and benign ovarian tumors using the 20% most varying probes and including biological replicates. n = 66 tumors.
(TIF)

Figure S2 **Hierarchical clustering.** Unsupervised clustering of malignant and benign ovarian tumors using the 20% most varying probes. Clustering performed without biological replicates. n = 54 tumors.
(TIF)

Figure S3 **Hierarchial clustering.** Supervised clustering of malignant, borderline and benign tumors based on significant probes from supervised analysis of malignant and benign tumors. Clustering performed without biological replicates. n = 59 tumors.
(TIF)

Figure S4 **Hierarchical clustering.** The serous ovarian tumors in the study cohort with corresponding tumor features and assigned ovarian cancer C-signatures and intrinsic breast cancer subtypes.
(TIF)

Figure S5 **Functional gene expression modules.** Boxplots representing the correlations between serous ovarian malignant, borderline and benign tumors in the study cohort and the respective gene expression modules by Desmedt *et al.* (Desmedt *et al.*, Clin Cancer Res 2008). Log2 mRNA values are presented on the Y axes. p-values are calculated using the Kruskal-Wallis test.
(TIF)

Figure S6 **Functional gene expression modules.** Boxplots representing the correlations between the C1-C6 ovarian cancer signatures in an independent, publicly available dataset and the respective breast cancer gene expression modules by Desmedt *et al.* (Desmedt *et al.*, Clin Cancer Res 2008). Log2 mRNA values are presented on the Y axes. p-values for the highlighted boxes vs. the rest in each plot are calculated using the Mann-Whitney U Test.
(TIF)

Table S1 **Deregulated genes.** All significantly deregulated genes (n = 5,944) between malignant and benign ovarian tumors in the study cohort.
(XLSX)

Table S2 **Molecular subtypes.** Significant correlations between malignant, borderline and benign ovarian tumors in the study cohort (n = 59) and the ovarian cancer C-signatures. p-values for each part of the table (separated with double lines) are calculated using Fisher's exact test.
(XLSX)

Table S3 **Molecular subtypes.** Significant correlations between malignant, borderline and benign ovarian tumors in the study cohort (n = 59) and the basal-like and normal-like breast cancer subtypes. p-values for each part of the table (separated with double lines) are calculated using Fisher's exact test.
(XLSX)

Table S4 **Molecular subtypes.** Significant correlations between malignant, borderline and benign ovarian tumors in the study cohort (n = 59) and the luminal A breast cancer subtype. The p-value is calculated using Fisher's exact test.
(XLSX)

Table S5 **Correlations between molecular subtypes.** Significant correlations between and between assigned C-signatures and the basal-like breast cancer subtype. p-values for each part of the table (separated with double lines) are calculated using Fisher's exact test.
(XLSX)

Table S6 **Correlations between molecular subtypes.** Significant correlations between and between assigned C-signatures and the normal-like breast cancer subtype. The p-value is calculated using Fisher's exact test.
(XLSX)

Table S7 **Validation of the centroid classifier.** Cross table comparing the original k.means groups (rows) for the tumors in an independent, publicly available dataset, consisting of malignant and borderline tumors, with the centroid classification for the same tumors (columns). The numbers represent number of tumors. Correlations between the C-signature classifications are highlighted in bold (diagonal). 251 tumors had an assigned k.means group. 239 of these (95.2%) were correctly re-assigned using nearest centroid classification.
(XLSX)

Table S8 **Correlations between molecular subtypes.** Correlations between ovarian cancer C-signatures and intrinsic breast cancer subtypes in an independent, publicly available dataset consisting of malignant and borderline ovarian tumors (n = 285). The C2 signature correlated significantly to the basal-like breast cancer subtype and the C3 signature to the normal-like breast cancer subtype. p-values (*) are calculated using Fisher's exact test.
(XLSX)

## References

[1] Kurman RJ, le Shih M (2010) The origin and pathogenesis of epithelial ovarian cancer: a proposed unifying theory. Am J Surg Pathol 34: 433–443. 20154587 10.1097/PAS.0b013e3181cf3d79 PMC2841791

[2] Piek JM, van Diest PJ, Zweemer RP, Jansen JW, Poort-Keesom RJ, et al (2001) Dysplastic changes in prophylactically removed Fallopian tubes of women predisposed to developing ovarian cancer. J Pathol 195: 451–456. 11745677 10.1002/path.1000

[3] Kindelberger DW, Lee Y, Miron A, Hirsch MS, Feltmate C, et al (2007) Intraepithelial carcinoma of the fimbria and pelvic serous carcinoma: Evidence for a causal relationship. Am J Surg Pathol 31: 161–169. 17255760 10.1097/01.pas.0000213335.40358.47

[4] Heintz AP, Odicino F, Maisonneuve P, Quinn MA, Benedet JL, et al (2006) Carcinoma of the ovary. FIGO 26th Annual Report on the Results of Treatment in Gynecological Cancer. Int J Gynaecol Obstet 95 Suppl 1 S161–192. 17161157 10.1016/S0020-7292(06)60033-7

[5] Bookman MA, Brady MF, McGuire WP, Harper PG, Alberts DS, et al (2009) Evaluation of new platinum-based treatment regimens in advanced-stage ovarian cancer: a Phase III Trial of the Gynecologic Cancer Intergroup. J Clin Oncol 27: 1419–1425. 19224846 10.1200/JCO.2008.19.1684 PMC2668552

[6] Winter-Roach BA, Kitchener HC, Dickinson HO (2009) Adjuvant (post-surgery) chemotherapy for early stage epithelial ovarian cancer. Cochrane Database Syst Rev: CD004706. 10.1002/14651858.CD004706.pub4 PMC4164914 22419298

[7] Perren TJ, Swart AM, Pfisterer J, Ledermann JA, Pujade-Lauraine E, et al (2011) A phase 3 trial of bevacizumab in ovarian cancer. N Engl J Med 365: 2484–2496. 22204725 10.1056/NEJMoa1103799

[8] McCluggage WG (2011) Morphological subtypes of ovarian carcinoma: a review with emphasis on new developments and pathogenesis. Pathology 43: 420–432. 21716157 10.1097/PAT.0b013e328348a6e7

[9] Saad AF, Hu W, Sood AK (2010) Microenvironment and pathogenesis of epithelial ovarian cancer. Horm Cancer 1: 277–290. 21761359 10.1007/s12672-010-0054-2 PMC3199131

[10] Prat J (2012) Ovarian carcinomas: five distinct diseases with different origins, genetic alterations, and clinicopathological features. Virchows Arch 460: 237–249. 22322322 10.1007/s00428-012-1203-5

[11] Kobel M, Kalloger SE, Boyd N, McKinney S, Mehl E, et al (2008) Ovarian carcinoma subtypes are different diseases: implications for biomarker studies. PLoS Med 5: e232. 19053170 10.1371/journal.pmed.0050232 PMC2592352

[12] Bonome T, Lee JY, Park DC, Radonovich M, Pise-Masison C, et al (2005) Expression profiling of serous low malignant potential, low-grade, and high-grade tumors of the ovary. Cancer Res 65: 10602–10612. 16288054 10.1158/0008-5472.CAN-05-2240

[13] Meinhold-Heerlein I, Bauerschlag D, Hilpert F, Dimitrov P, Sapinoso LM, et al (2005) Molecular and prognostic distinction between serous ovarian carcinomas of varying grade and malignant potential. Oncogene 24: 1053–1065. 15558012 10.1038/sj.onc.1208298

[14] Marquez RT, Baggerly KA, Patterson AP, Liu J, Broaddus R, et al (2005) Patterns of gene expression in different histotypes of epithelial ovarian cancer correlate with those in normal fallopian tube, endometrium, and colon. Clin Cancer Res 11: 6116–6126. 16144910 10.1158/1078-0432.CCR-04-2509

[15] Marchini S, Mariani P, Chiorino G, Marrazzo E, Bonomi R, et al (2008) Analysis of gene expression in early-stage ovarian cancer. Clin Cancer Res 14: 7850–7860. 19047114 10.1158/1078-0432.CCR-08-0523

[16] The Cancer Genome Atlas Network (2011) Integrated genomic analyses of ovarian carcinoma. Nature 474: 609–615. 21720365 10.1038/nature10166 PMC3163504

[17] Yoshihara K, Tsunoda T, Shigemizu D, Fujiwara H, Hatae M, et al (2012) High-risk ovarian cancer based on 126-gene expression signature is uniquely characterized by downregulation of antigen presentation pathway. Clin Cancer Res 18: 1374–1385. 22241791 10.1158/1078-0432.CCR-11-2725

[18] Tothill RW, Tinker AV, George J, Brown R, Fox SB, et al (2008) Novel molecular subtypes of serous and endometrioid ovarian cancer linked to clinical outcome. Clin Cancer Res 14: 5198–5208. 18698038 10.1158/1078-0432.CCR-08-0196

[19] The Cancer Genome Atlas Network (2012) Comprehensive molecular portraits of human breast tumours. Nature 490: 61–70. 23000897 10.1038/nature11412 PMC3465532

[20] Perou CM, Sorlie T, Eisen MB, van de Rijn M, Jeffrey SS, et al (2000) Molecular portraits of human breast tumours. Nature 406: 747–752. 10963602 10.1038/35021093

[21] Sorlie T, Perou CM, Tibshirani R, Aas T, Geisler S, et al (2001) Gene expression patterns of breast carcinomas distinguish tumor subclasses with clinical implications. Proc Natl Acad Sci U S A 98: 10869–10874. 11553815 10.1073/pnas.191367098 PMC58566

[22] Hu Z, Fan C, Oh DS, Marron JS, He X, et al (2006) The molecular portraits of breast tumors are conserved across microarray platforms. BMC Genomics 7: 96. 16643655 10.1186/1471-2164-7-96 PMC1468408

[23] Anglesio MS, Arnold JM, George J, Tinker AV, Tothill R, et al (2008) Mutation of ERBB2 provides a novel alternative mechanism for the ubiquitous activation of RAS-MAPK in ovarian serous low malignant potential tumors. Mol Cancer Res 6: 1678–1690. 19010816 10.1158/1541-7786.MCR-08-0193 PMC6953412

[24] Shih Ie M, Kurman RJ (2004) Ovarian tumorigenesis: a proposed model based on morphological and molecular genetic analysis. Am J Pathol 164: 1511–1518. 15111296 10.1016/s0002-9440(10)63708-x PMC1615664

[25] Silverberg SG (2000) Histopathologic grading of ovarian carcinoma: a review and proposal. Int J Gynecol Pathol 19: 7–15. 10638449 10.1097/00004347-200001000-00003

[26] Tavassoli FA, Devilee P (2003) Pathology and Genetics of Tumours of the Breast and Female Genital Organs; Tavassoli Fattaneh A DP, editor. Lyon: IARC Press.

[27] Edgar R, Domrachev M, Lash AE (2002) Gene Expression Omnibus: NCBI gene expression and hybridization array data repository. Nucleic Acids Res 30: 207–210. 11752295 10.1093/nar/30.1.207 PMC99122

[28] Saeed AI, Sharov V, White J, Li J, Liang W, et al (2003) TM4: a free, open-source system for microarray data management and analysis. Biotechniques 34: 374–378. 12613259 10.2144/03342mt01

[29] Tusher VG, Tibshirani R, Chu G (2001) Significance analysis of microarrays applied to the ionizing radiation response. Proc Natl Acad Sci U S A 98: 5116–5121. 11309499 10.1073/pnas.091062498 PMC33173

[30] Eden E, Navon R, Steinfeld I, Lipson D, Yakhini Z (2009) GOrilla: a tool for discovery and visualization of enriched GO terms in ranked gene lists. BMC Bioinformatics 10: 48. 19192299 10.1186/1471-2105-10-48 PMC2644678

[31] Chen J, Aronow BJ, Jegga AG (2009) Disease candidate gene identification and prioritization using protein interaction networks. BMC Bioinformatics 10: 73. 19245720 10.1186/1471-2105-10-73 PMC2657789

[32] Johansson I, Nilsson C, Berglund P, Lauss M, Ringner M, et al (2012) Gene expression profiling of primary male breast cancers reveals two unique subgroups and identifies N-acetyltransferase-1 (NAT1) as a novel prognostic biomarker. Breast Cancer Res 14: R31. 22333393 10.1186/bcr3116 PMC3496149

[33] Desmedt C, Haibe-Kains B, Wirapati P, Buyse M, Larsimont D, et al (2008) Biological processes associated with breast cancer clinical outcome depend on the molecular subtypes. Clin Cancer Res 14: 5158–5165. 18698033 10.1158/1078-0432.CCR-07-4756

[34] Hauptmann S, Dietel M (2001) Serous tumors of low malignant potential of the ovary-molecular pathology: part 2. Virchows Arch 438: 539–551. 11469685 10.1007/s004280100435

[35] Li J, Fadare O, Xiang L, Kong B, Zheng W (2012) Ovarian serous carcinoma: recent concepts on its origin and carcinogenesis. J Hematol Oncol 5: 8. 22405464 10.1186/1756-8722-5-8 PMC3328281

[36] Berns EM, Bowtell DD (2012) The changing view of high-grade serous ovarian cancer. Cancer Res 72: 2701–2704. 22593197 10.1158/0008-5472.CAN-11-3911

[37] Perez-Gracia JL, Carrasco EM (2002) Tamoxifen therapy for ovarian cancer in the adjuvant and advanced settings: systematic review of the literature and implications for future research. Gynecol Oncol 84: 201–209. 11812075 10.1006/gyno.2001.6489

[38] Curry EW, Stronach EA, Rama NR, Wang YY, Gabra H, et al. (2013) Molecular subtypes of serous borderline ovarian tumor show distinct expression patterns of benign tumor and malignant tumor-associated signatures. Mod Pathol. 10.1038/modpathol.2013.130 23948749

[39] Smyth JF, Gourley C, Walker G, MacKean MJ, Stevenson A, et al (2007) Antiestrogen therapy is active in selected ovarian cancer cases: the use of letrozole in estrogen receptor-positive patients. Clin Cancer Res 13: 3617–3622. 17575226 10.1158/1078-0432.CCR-06-2878

[40] Hatch KD, Beecham JB, Blessing JA, Creasman WT (1991) Responsiveness of patients with advanced ovarian carcinoma to tamoxifen. A Gynecologic Oncology Group study of second-line therapy in 105 patients. Cancer 68: 269–271. 2070324 10.1002/1097-0142(19910715)68:2<269::aid-cncr2820680209>3.0.co;2-o

[41] Chan KK, Wei N, Liu SS, Xiao-Yun L, Cheung AN, et al (2008) Estrogen receptor subtypes in ovarian cancer: a clinical correlation. Obstet Gynecol 111: 144–151. 18165403 10.1097/01.AOG.0000296715.07705.e9

[42] Burges A, Bruning A, Dannenmann C, Blankenstein T, Jeschke U, et al (2010) Prognostic significance of estrogen receptor alpha and beta expression in human serous carcinomas of the ovary. Arch Gynecol Obstet 281: 511–517. 19639330 10.1007/s00404-009-1185-y

[43] Early Breast Cancer Trialists' Collaborative Group (2005) Effects of chemotherapy and hormonal therapy for early breast cancer on recurrence and 15-year survival: an overview of the randomised trials. Lancet 365: 1687–1717. 15894097 10.1016/S0140-6736(05)66544-0

[44] Sieh W, Kobel M, Longacre TA, Bowtell DD, deFazio A, et al (2013) Hormone-receptor expression and ovarian cancer survival: an Ovarian Tumor Tissue Analysis consortium study. Lancet Oncol 14: 853–862. 23845225 10.1016/S1470-2045(13)70253-5 PMC4006367

