# Supplementary figures and images for: Molecular Subtyping of Serous Ovarian Tumors Reveals Multiple Connections to Intrinsic Breast Cancer Subtypes
Source: PLoS One. 2014 Sep 16;9(9):e107643. doi: 10.1371/journal.pone.0107643 (PMC4166462; doi:10.1371/journal.pone.0107643)

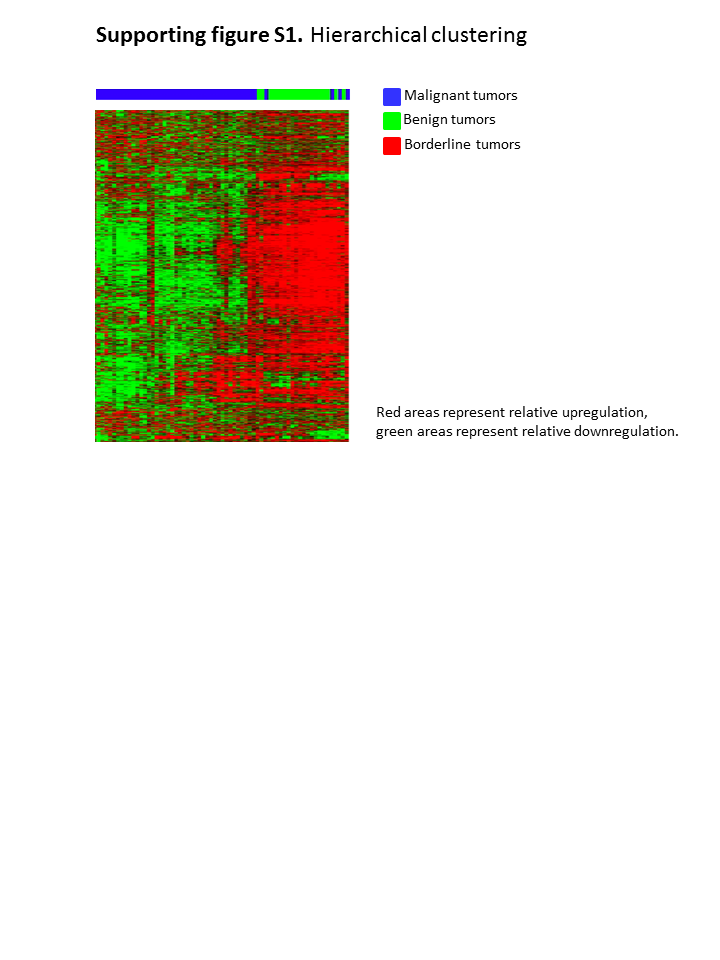

Supplement: Figure S1 — Hierarchical clustering. Unsupervised clustering of malignant and benign ovarian tumors using the 20% most varying probes and including biological replicates. n = 66 tumors. (TIF) [file pone.0107643.s001.tif]

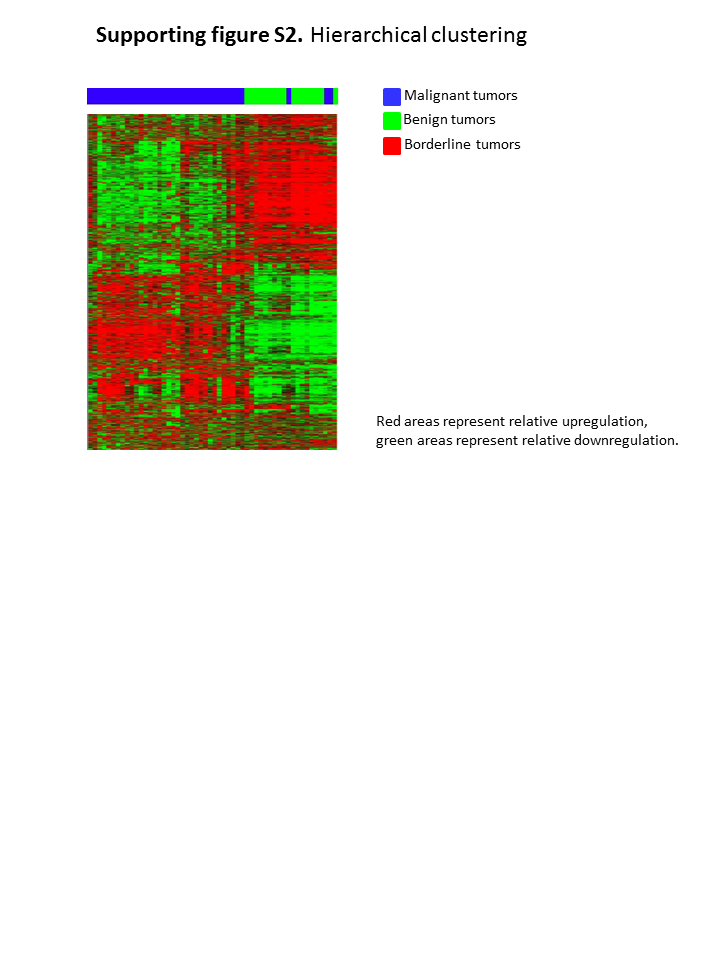

Supplement: Figure S2 — Hierarchical clustering. Unsupervised clustering of malignant and benign ovarian tumors using the 20% most varying probes. Clustering performed without biological replicates. n = 54 tumors. (TIF) [file pone.0107643.s002.tif]

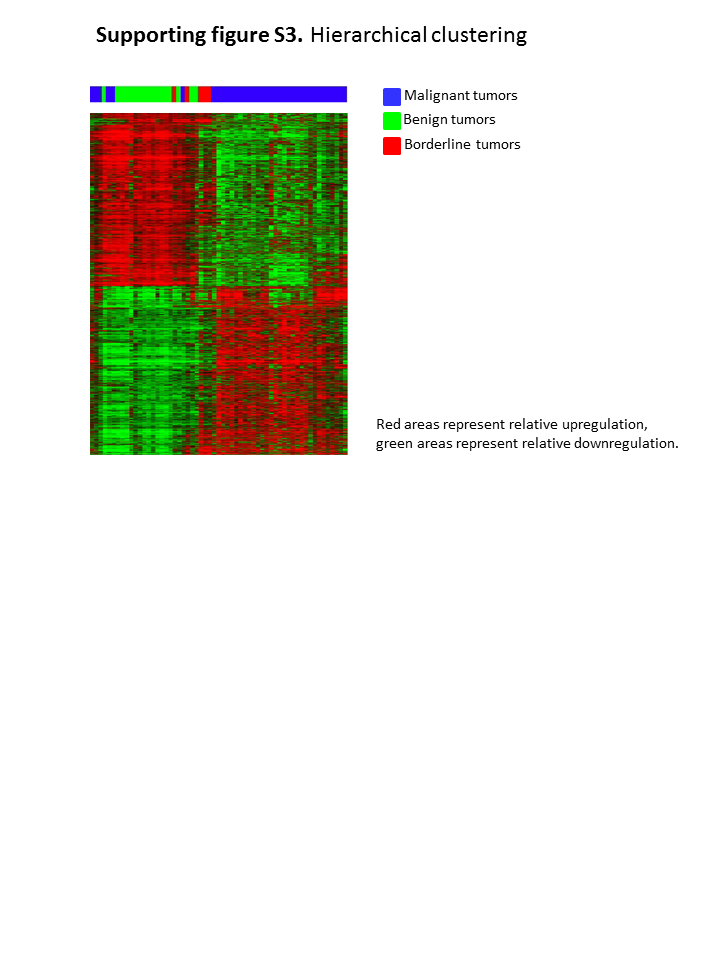

Supplement: Figure S3 — Hierarchial clustering. Supervised clustering of malignant, borderline and benign tumors based on significant probes from supervised analysis of malignant and benign tumors. Clustering performed without biological replicates. n = 59 tumors. (TIF) [file pone.0107643.s003.tif]

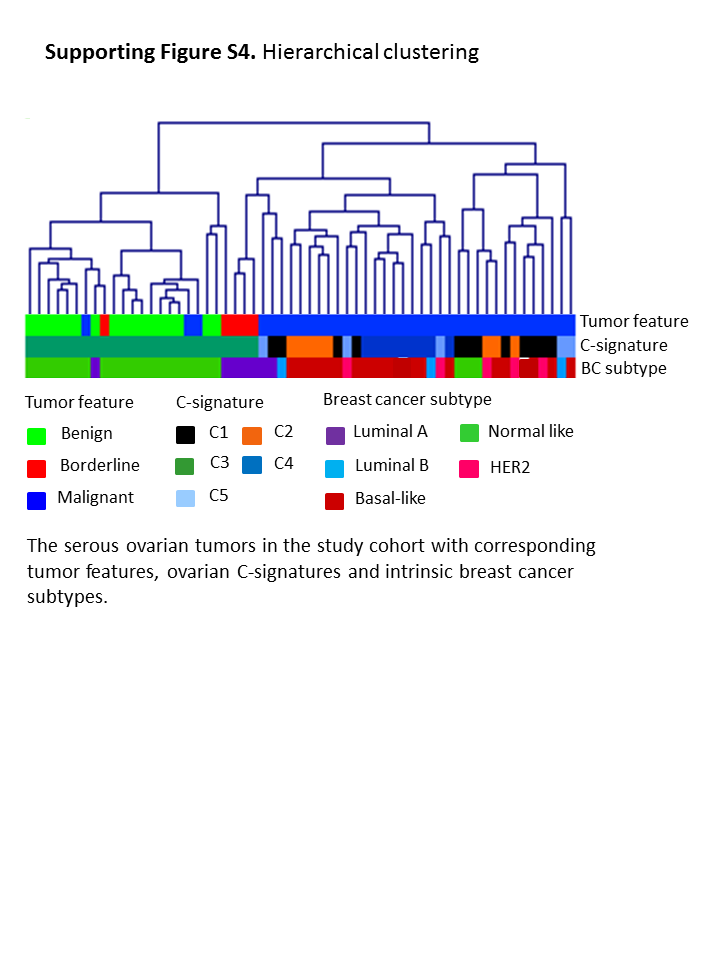

Supplement: Figure S4 — Hierarchical clustering. The serous ovarian tumors in the study cohort with corresponding tumor features and assigned ovarian cancer C-signatures and intrinsic breast cancer subtypes. (TIF) [file pone.0107643.s004.tif]
